# Neutropenic Fever in a Patient With SARS-CoV-2-Induced Hemophagocytic Lymphohistiocytosis (HLH)

**DOI:** 10.7759/cureus.20101

**Published:** 2021-12-02

**Authors:** Elliot Runge, Steven Stoffel, Matthew Rendo, Bradley W Beeler

**Affiliations:** 1 Internal Medicine, Brooke Army Medical Center, Fort Sam Houston, USA; 2 Hematology and Oncology, Brooke Army Medical Center, Fort Sam Houston, USA

**Keywords:** covid-19, hemophagocytic lymphohistiocytosis (hlh), neutropenic fever, non hodgkin's lymphoma, cytokine release storm

## Abstract

Hemophagocytic lymphohistiocytosis (HLH) is a severe systemic inflammatory syndrome that is often fatal. In the adult population, it is believed to develop secondary to immune dysregulation due to rheumatologic, infectious, malignant, and recently, immunomodulatory drugs. It has been well documented that infectious etiologies can lead to HLH however to date there is a paucity of case reports of HLH stemming from the 2019 novel coronavirus (SARS-CoV-2). Furthermore, it is well established that overlap exists between the extensive hyper-inflammatory syndromes produced from both HLH and severe COVID-19 infection. Here, we present a case of COVID-19-associated HLH with recurrent neutropenic fever in a patient with controlled follicular non-Hodgkin lymphoma who received treatment with etoposide after continued hospital admissions with refractory medical treatment.

## Introduction

Hemophagocytic lymphohistiocytosis (HLH) is a hostile immune hyper-activation and hyper-inflammatory syndrome which often includes a common terminal pathway triggered by a wide variety of etiologies. Phenotypically, HLH results in life-threatening inflammation, hypercytokinemia, hemophagocytosis and multi-organ failure [1,2]. The disease is defined by a reduction in the manufacture of interferon-gamma production with macrophage phagocytosis of erythrocytes, leukocytes, platelets and cellular precursors in the bone marrow and other tissue which shares pathophysiology to other hyper-inflammatory syndromes. HLH can be categorized as either a primary or a secondary syndrome [1-3]. Primary hemophagocytosis usually presents in early childhood and results in cytotoxic impairment in natural killer (NK) cells [1,2]. Secondary HLH is associated with predisposing conditions, such as rheumatologic, malignant or infectious etiologies.

Viral etiologies are known to be linked to the development of secondary HLH and other hemophagocytic syndromes [4-6]. Epstein-Barr virus (EBV) and herpes simplex virus (HSV) infections are frequent causes of secondary HLH; however other viruses (e.g., cytomegalovirus, hepatitis A, parvovirus B19) and pathogens have also been implicated [7]. There is growing evidence supporting a similar pathophysiologic mechanism between COVID disease and HLH, which includes a hyperactive immune response with a triggered cytokine storm. Recent case reports continue to demonstrate a correlation in COVID-mediated HLH which further supports a link between their similar pathophysiologic propagation. Therefore, SARS-CoV-2, as other respiratory viruses, may also be considered a potential etiological trigger of HLH [8].

## Case presentation

A 38-year-old male with a medical history of stage IVB follicular non-Hodgkin lymphoma presented to the hospital with fevers to 103F and was found to have severe neutropenia to 180 cells/microL. Of note, the patient’s lymphoma had previously been treated and achieved remission after six cycles of bendamusine (alkylating agent) and obinutuzumab (anti-CD-20 monoclonal antibody). At the time of presentation, the patient was on maintenance obinutuzumab with plans for an eventual bone marrow transplant; however, his course was complicated by repeated bouts of severe neutropenia requiring administration of filgrastim (G-CSF) to maintain neutrophil count.

In addition to developing fevers to 103F at home, the patient also had a cough, non-bloody emesis, anosmia and inability to tolerate oral fluids which prompted his presentation to the emergency department. One week prior to presentation, the patient had tested positive for SARS-COVID-19 for which the only symptoms he manifested at that time was anosmia. On initial presentation he was febrile but hemodynamically stable and not requiring any supplemental oxygen; initial labs showed an absolute neutrophil count of 180 cells/microL, slight normocytic anemia, mild acute kidney injury and mild hyponatremia. Full infectious workup was obtained and the patient was placed on appropriate broad-spectrum antibiotics. After an extensive infectious workup for bacterial, fungal and viral etiologies, only the known SARS-COVID-19 infection was identified as the potential trigger for the neutropenic fever. After a six-day hospital stay, the patient had significant improvement in symptoms and improvement of neutrophil count following filgrastim administrations. The patient was discharged home with recommendations for close follow-up.

One week following discharge patient was represented to the Emergency department due to three days of fevers, rigors and malaise. The patient was additionally experiencing coughing spells with post-tussive emesis. The patient was febrile on presentation with hypotension meeting sepsis criteria. Sepsis protocol was initiated including a collection of blood cultures, bolus intravenous fluids and starting of empiric antibiotics. Initial labs were significant for an absolute neutrophil count of 220 cells/microL, lactate of 1.9 and elevated creatinine of 1.4 (baseline 1.0). The patient again tested positive for SARS-COVID-19. The patient was stabilized in the Emergency Department and again admitted to the hospital.

During his hospital course #2, the patient continued to fever with rigors and gradually worsening respiratory status. Antibiotics were broadened to cover bacterial and fungal etiologies, with empiric antifungal coverage. Blood cultures continued to return with no evidence of bacterial infection and screening for fungal etiology (Fungitell and Galactomannan) were also negative. The patient had developed a new oxygen requirement and computed tomography of the chest showed worsening multilobar opacities, concerning an evolving progressive viral pneumonia. During hospitalization, the patient’s absolute neutrophil count gradually decreased to <500 cells/microL, and filgrastim was restarted to maintain ANC > 300 cells/microL. Due to the lack of infectious etiology on screening labs, a decision was made to perform bronchoscopy with bronchoalveolar lavage. Results showed normal respiratory flora, with no evidence of fungal infection. Respiratory status continued to worsen and the patient required up to 40L of supplemental oxygen via a high flow nasal cannula. The patient was subsequently transferred to the Medical Intensive Care Unit due to declining respiratory status.

**Figure 1:**
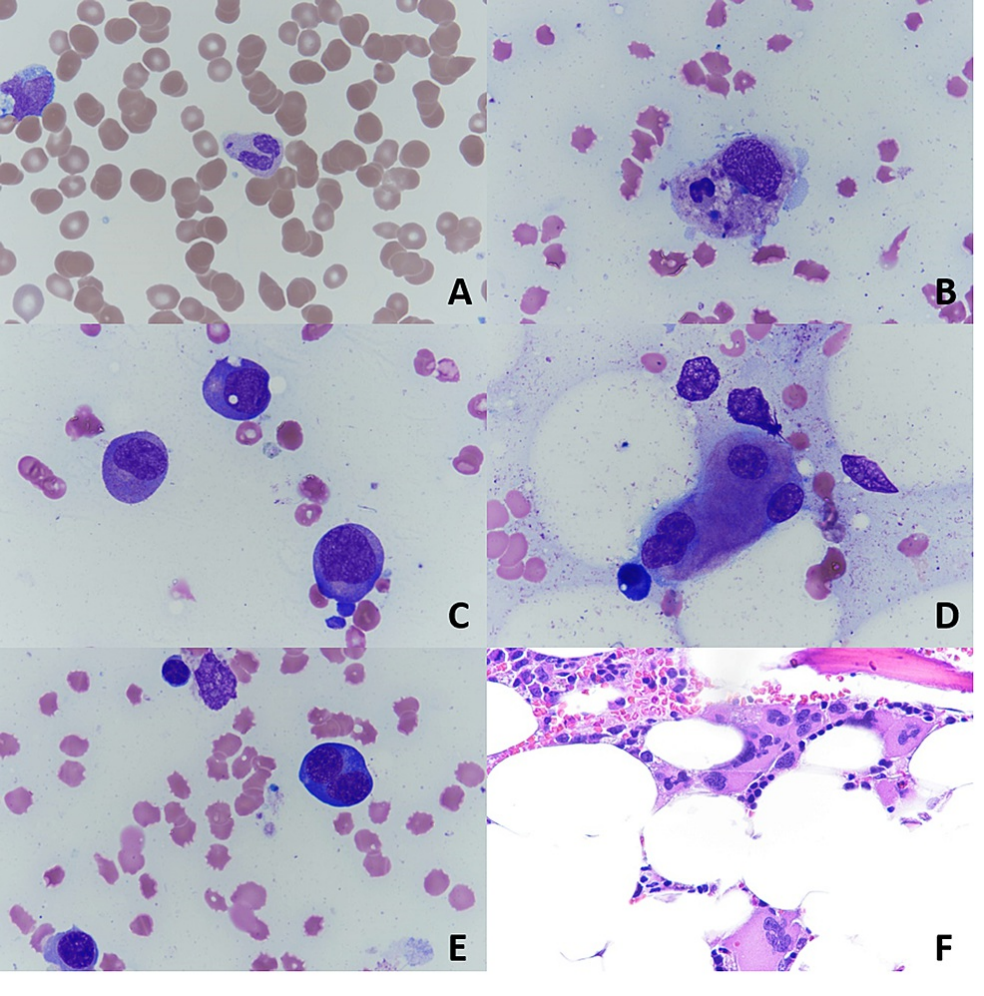
Peripheral smear demonstrates granulocyte dysplasia with hypogranular and hyposegmented neutrophils with detached nuclear fragments (A). Bone marrow aspirate demonstrates hemophagocytosis (B), nuclear-cytoplasmic dyssynchrony (C), megakaryocyte dysplasia (D) and <10% erythroid dysplasia (E). Bone marrow core biopsy demonstrates clustering of dysplastic megakaryocytes and myeloid maturation arrest (F). (A-E) Wright Giemsa stain, 100x oil immersion lens. (F) Hematoxylin and Eosin stain, 40x objective lens.

After discussion with Hematology/Oncology, a bone marrow biopsy was performed due to progressive pancytopenia, rising ferritin of 2500 and elevated CRP. Bone marrow biopsy demonstrated excessive macrophage-mediated inflammatory response, consistent with HLH (Figures 1A-1F). Additional laboratory analysis including NK cell activity and CD25 levels corresponded to an H-score of 216 which was consistent with a 93% probability of hemophagocytic syndrome. The patient met six out of eight criteria to make definitive diagnosis of HLH: fever > 102.9F, AST > 30, trilineage cytopenia, fasting triglycerides > 265 mg/dL, hemophagocytosis in bone marrow, ferritin > 500 ng/mL and elevated soluble CD25 (IL-2 receptor). The patient was started on high-dose dexamethasone for presumed HLH secondary to SARS-COVID-19 infection. After starting high dose dexamethasone (24 mg daily) patient’s clinical status quickly improved with a decrease in fevers and oxygen requirements. The patient was eventually transferred out of the Medical ICU with continued improvement in serum ferritin; his oxygen was eventually weaned to room air and was discharged home on a prolonged steroid taper and close follow-up.

**Figure 2:**
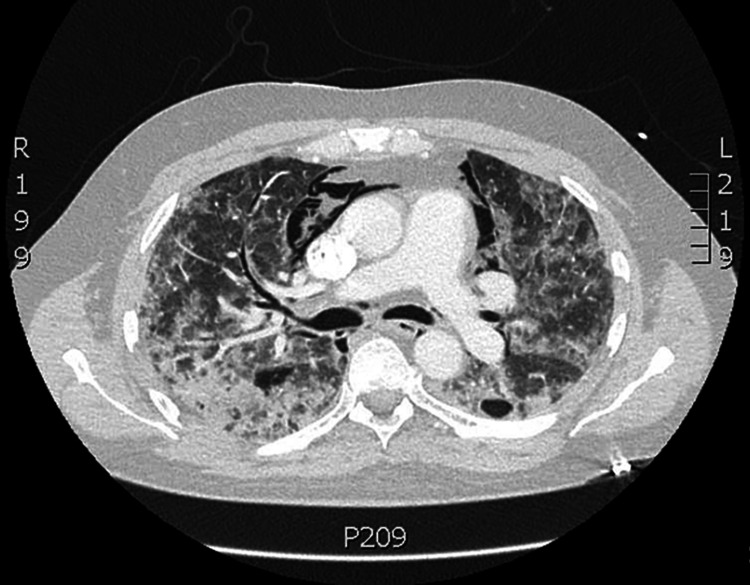
Extensive pneumomediastinum which has progressed which appears to exert some mass effect on the right ventricle. Diffuse bilateral ground-glass and consolidative opacities consistent with a history of COVID pneumonia.

One month following discharge, the patient again presented to the Emergency Department for acute onset neck pain, difficulty swallowing and worsening shortness of breath. The patient was hemodynamically stable but was admitted for hospitalization #3 for hypoxemic respiratory failure requiring supplemental oxygen of 15L via OxyMask. CT neck and chest performed significantly for pneumomediastinum with soft tissue emphysema surrounding carotid arteries (Figure 2). Neutropenia was again observed during this hospitalization to 50 cells/microL with continued fevers and lactate to 4.0. Repeat infectious workup was completed and the patient again started on empiric antibiotics. Inflammatory markers were found to be persistently elevated, concerning persistent/recurrent HLH. Repeat bone marrow biopsy was again performed and interestingly was without evidence of hemophagocytosis. Despite this, the patient did meet 5/8 diagnostic criteria for HLH, meeting criteria for diagnosis. The clinical suspicion for recurrent/refractory HLH was high, as such dexamethasone was increased.

Despite treatment with increased steroid dose, the patient’s clinical course continued to worsen to include airway compromise requiring endotracheal intubation and hypotension requiring vasopressor support. Additional treatment including IVIG and tocilizumab was completed. The hospital course was further complicated by ventilator-associated pneumonia (Pseudomonas spp.), renal failure requiring renal replacement therapy and subsequent development of severe GI bleeding, requiring multiple blood transfusions. Due to unsuccessful response to management, treatment of HLH was expanded by adding etoposide (following HLH-2004 protocol). Despite this, the patient’s clinical status continued to deteriorate. Ventilator support continued to increase and the patient became difficult to ventilate, requiring increased positive pressure support. Hypotension continued to worsen even with the up-titration of vasopressors. The patient subsequently had several episodes of cardiac arrest secondary to hypoxia and hypotension. After four episodes of cardiac arrest, the family opted to discontinue further life-saving measures. The patient subsequently expired following the discontinuation of life-prolonging treatment. A primary cause of death is listed as HLH secondary to SARS-COVID-19 infection.

## Discussion

HLH is not simply a solitary disease but rather an expansive clinical syndrome characterized by excessive activation of lymphocytes and macrophages resulting in elevated cytokine levels. To adequately establish a diagnosis of HLH, five of eight diagnostic criteria must be made which come from the modified 2009 HLH criteria. HLH secondary to underlying genetic aberrations is known as primary HLH. Primary HLH is further divided into either familial HLH or immune deficiencies such as X-linked lymphoproliferative syndrome (XLP) or Griscelli syndrome (GS) [1,5].

HLH occurring secondary to other related diseases (secondary HLH) due to underlying malignancy, autoimmune phenomena, or strong infectious syndrome represent a significant subset of diseases. Infectious-based secondary HLH has been shown extensively in case series with viral infections such as influenza, EBV and cytomegalovirus (CMV) [4]. Other common infectious etiologies for secondary HLH include but are not limited to human immunodeficiency virus (HIV), Mycobacterium tuberculosis, rickettsia, leishmaniasis and histoplasmosis [9]. Possibly the most well-established viral etiology of infection-related secondary HLH is EBV which incidence ranging from 33% to 75% of HLH patients [10,11]. However, there exists a growing amount of case series demonstrating a clear association with the SARS-CoV-2 viral as a potential cause of infection-related secondary HLH [12]. The mechanism behind this is owed in part to the profound cytokine storm that is propagated by a hyper-inflammatory state that severe COVID infections can produce [13]. Prior studies have suggested that SARS-CoV-2 could activate NLRP3 inflammasomes that active macrophages, resulting in an increased release of IL-1β, which subsequently contributes to IL-6 release [14]. The possibility of other viruses contributing to a hyper-inflammatory hemophagocytic state should be entertained particularly with viruses that stimulate significant cytokine storms such as COVID. Taken together, it is very reasonable to consider COVID viral infections as a clear contributing factor for secondary HLH.

In this patient, the leading pathophysiologic mechanism thought to be responsible for the propagation of secondary HLH was severe SARS-COVID-19 infection in the setting of baseline neutropenia secondary to six cycles of bendamustine (alkylating agent) and maintenance obinutuzumab for his stage IVB follicular non-Hodgkin lymphoma. Obinutuzumab is an anti-CD20 monoclonal antibody and while its main effect is on B-cell neoplasms, there do exist side effects of myelosuppression specifically neutropenia in 22%-59% when used as monotherapy which was likely one of the causes of the patient's prolonged neutropenia prior to his initial presentation [15]. COVID-19 infection on its own results not only in a global hyper-inflammatory state (similar to that of HLH) but also can be exacerbated in patients with a prolonged depletion of B-lymphocytes [14]. Therefore it is reasonable to assume that in our patient there was an already existent B-lymphocyte depleted environment with concomitant neutropenia with which a superimposed COVID-19 infection served as a precipitant in the development of the hyper-inflammatory hemophagocytic state. One can also extrapolate this thought in our patient’s case to assume that all his subsequent hospital course complications were in some way related to his developing HLH and his underlying neutropenia. Other theories were considered regarding why this patient developed HLH; however, it is interesting to note that in both bone marrow biopsies performed there was no evidence of reoccurrence of his prior non-Hodgkin lymphoma, therefore, making a malignancy etiology less likely.

Various treatment regimens were attempted in this patient such as tocilizumab and etoposide. Etoposide specifically has been shown to increase long-term survival in patients with EBV-associated secondary HLH as has now made its way to standard of care per the HLH-94/04 regimen [16,17]. However, these regimens in the HLH -94/04 protocol have not been studied especially in SARS-COVID-19-associated secondary HLH and highlight the clinical relevance and research question which needs to be addressed with future studies. Despite the initial treatment response our patient had with etoposide and tocilizumab, complications arose in subsequent hospitalizations that lead to the patient’s demise however this should not deter other practitioners from adhering to a standard of care for all secondary HLH.

## Conclusions

HLH is considered a syndrome of excess inflammation due to dysregulated immune modulation. Hemophagocytosis of red blood cells, platelets and/or white blood cells is seen pathophysiologically. Since the start of the 2020 pandemic, the potential of SARS-CoV-2 leading to hyperinflammatory response and subsequent HLH is being seen more and more which suggests a link between the cytokine storm seen both in HLH and in SARS-CoV-2. There is a growing body of case reports not only suggesting this link in the similar pathophysiology between HLH and SARS-CoV-2 but also perhaps severe COVID infection-causing secondary HLH, which is what was seen in this case. With time, it is likely that more hyper-inflammatory, immunomodulatory and hemophagocytic associations will be made with severe COVID infections. Perhaps more importantly, due to the similarity between these pathophysiologic states, there could exist similar treatment strategies aimed at curtailing the hyperinflammatory response seen in these diseases which leads to a patient's increased mortality. Therefore, more research is needed in this realm to develop treatment methods aimed at targeting the inflammatory response.
